# How participants report their health status: cognitive interviews of self-rated health across race/ethnicity, gender, age, and educational attainment

**DOI:** 10.1186/s12889-017-4761-2

**Published:** 2017-10-04

**Authors:** Dana Garbarski, Jennifer Dykema, Kenneth D. Croes, Dorothy F. Edwards

**Affiliations:** 1 Department of Sociology, Loyola University Chicago, Coffey Hall 440, 1032 W. Sheridan Rd, Chicago, IL 60660 USA; 2 University of Wisconsin Survey Center, University of Wisconsin-Madison, 475 N. Charter Street, Room 4308, Madison, WI 53706 USA; 3 University of Wisconsin Survey Center, University of Wisconsin-Madison, 475 N. Charter Street, Room 4416, Madison, WI 53706 USA; 4 Departments of Kinesiology-Occupational Therapy Program, Neurology and Medicine, University of Wisconsin-Madison, 2170 Medical Science Center, 1300 University Avenue, Madison, WI 53706-1532 USA

**Keywords:** US, Self-rated health, Cognitive interviewing, Grounded theory coding, Evaluative frameworks, Response process, Health disparities, Sociodemographic differences

## Abstract

**Background:**

Self-rated health (SRH) is widely used to measure subjective health. Yet it is unclear what underlies health ratings, with implications for understanding the validity of SRH overall and across sociodemographic characteristics. We analyze participants’ explanations of *how* they formulated their SRH answer in addition to *which* health factors they considered and examine group differences in these processes.

**Methods:**

Cognitive interviews were conducted with 64 participants in a convenience quota sample crossing dimensions of race/ethnicity (white, Latino, black, American Indian), gender, age, and education. Participants rated their health then described their thoughts when answering SRH. We coded participants’ answers in an inductive, iterative, and systematic process from interview transcripts, developing analytic categories (i.e., themes) and subdimensions within. We examined whether the presence of each dimension of an analytic category varied across sociodemographic groups.

**Results:**

Our qualitative analysis led to the identification and classification of various subdimensions of the following analytic categories: types of health factors mentioned, valence of health factors, temporality of health factors, conditional health statements, and descriptions and definitions of health. We found differences across groups in some types of health factors mentioned—corresponding, conflicting, or novel with respect to prior research. Furthermore, we also documented various processes through which respondents integrate seemingly disparate health factors to formulate an answer through valence and conditional health statements. Finally, we found some evidence of sociodemographic group differences with respect to types of health factors mentioned, valence of health factors, and conditional health statements, highlighting avenues for future research.

**Conclusion:**

This study provides a description of how participants rate their general health status and highlights potential differences in these processes across sociodemographic groups, helping to provide a more comprehensive understanding of how SRH functions as a measure of health.

## Background

### Previous studies of the health factors underlying SRH

Self-rated health (SRH)—“Would you say your health in general is excellent, very good, good, fair, or poor?”—is one of the most widely used measures of health in social and behavioral sciences survey research, sometimes as the only or one of a few measures of health. In addition to its use in academic research, SRH is also used as summary indicator to monitor the health of populations [1] and patients in clinical settings [2]. The popularity of SRH stems in part from its predictive validity—the ability of this one survey measure to predict morbidity and mortality [3]. The lack of specificity in SRH is both its most important benefit and drawback, as respondents can holistically summarize their health, yet researchers cannot control which health factors respondents consider when rating their health [4].

Because of its ubiquity, utility, and lack of specificity, studies spanning several disciplines and decades attempt to uncover what factors participants consider when they provide a global rating of their health in a survey. The approach of these studies stems from two distinct methodological perspectives. First are quantitatively-focused studies that examine associations between SRH and other domains of health (e.g., such as questions about chronic conditions or health behaviors that are chosen a priori by the researcher). These studies draw inferences about which domains are considered more thoroughly by participants when rating their health based on which factors are most strongly associated with SRH ratings (see Garbarski [4] for a review). In contrast are more qualitatively-focused studies that ascertain the types of health factors participants take into account when asked to rate their health. Some of these studies use cognitive interviewing techniques in which participants rate their health and then answer follow-up questions about what they were thinking of while answering [5–9]. Other studies use semi-structured interviewing protocols [10, 11]. The qualitative studies often use a grounded theory approach in which codes, categories, and themes are identified simultaneously during an inductive and iterative coding process [12–14]. Because they emerge directly from talk produced by participants, the types of health factors reported by participants in qualitative interviews may be more representative of the participants’ lived experiences than variables chosen a priori.

The different qualitative studies offer several insights into participants’ interpretations of health. First, considered together, the qualitatively focused studies delineate the breadth of health factors participants use to construct their health rating, including health conditions, health behaviors, physical functioning factors, internal feelings or sensations, access to health care, mental and spiritual health, coping with illness, comparisons to others, situational factors, and even the context of prior questions [5–11]. Second, these studies provide evidence that the types of health factors participants consider often vary based on the response category (e.g., “excellent,” “very good,” “good,” “fair,” or “poor”) participants choose to classify their health [6–8, 11]. Third, in-depth analysis of qualitative responses highlight substantial variation in the nature of the health factors considered for a given response category [5]. Our study similarly seeks to contribute to this line of inquiry by exploring the types of health factors participants consider when rating their health across a diverse sample of participants.

However, the prior research on what underlies SRH answers is limited by its focus on *which* health factors participants consider when rating their health, aggregating responses into categories such as “health conditions,” “health behaviors,” and the like. Although informative, focusing on health factors alone says little about *how* participants take health factors into account when rating their health: a description of the different ways in which health factors and components of the SRH question are experienced, conceptualized, interpreted, and integrated to formulate health ratings. Review articles on SRH [3, 4, 15] have repeatedly called for more qualitative research to elucidate features of the SRH response process: comprehension of the question, retrieval of relevant information from memory to answer the question, use of retrieved information to make judgments, and selection and reporting of an answer [15, 16]. The current study seeks to address in part this gap in the literature.

### Variation in rating health across sociodemographic characteristics

In the course of documenting the various health factors that participants consider when they answer SRH, some studies suggest that responses to SRH vary across sociodemographic characteristics—such as race or ethnicity, gender, age, and socioeconomic status—among individuals that are otherwise similarly situated with respect to more objective measures of health (see Garbarski [4] for a review). These systematic differences across groups in the association between SRH and more objective health measures highlight that SRH answers constitute both the health factors participants consider when rating their health as well as the frameworks through which participants evaluate and rate their health [4, 15]. The notion of frameworks acknowledges that SRH answers are comprised of and influenced by a variety of social and psychological factors that provide a lens through which the health factors in one’s life and components of the question are considered when one is asked to rate their health [4, 15]. Frameworks operate consciously and unconsciously, influencing implicit definitions of health, referents used for making comparisons, types and scope of health factors considered, and interpretations of the survey question and response options [4, 15]. Overall, prior research documents that SRH answers vary across social groups with seemingly similar objective health characteristics, but no study has examined whether *how* participants take health factors into account when rating their health may vary across sociodemographic characteristics, an important first step in examining how evaluative framework differences across groups inform differences in SRH across those groups [3, 4, 15].

### Current study

Our study seeks to provide a more comprehensive understanding of how SRH functions as a measure of health in two ways. First, we examine the processes participants go through to formulate a judgment about the overall quality of their health that includes both *which* types of health factors participants take into account as well as *how* participants take these health factors and components of the SRH question into account when rating their health. This examination highlights the different ways in which health factors and components of the SRH question are experienced, conceptualized, interpreted, and integrated to formulate health ratings. Second, we are able to describe the ways in which these processes might vary across several important sociodemographic characteristics, including race/ethnicity, gender, age, and educational attainment, given our uniquely structured sample.

## Methods

### Cognitive interviewing protocol

We used a cognitive interviewing protocol to observe how participants formulate health ratings. The survey opened with the SRH question (adopted from the 2013 National Health Interview Survey): “Would you say your health in general is excellent, very good, good, fair, or poor?” A series of open-ended probes then followed to uncover how participants arrived at their answer. These probes, which included “What were you thinking about when you answered (ANSWER) for this question” and “What else were you thinking about” sought to reveal any problems participants had with comprehension of specific terms, retrieval of information from memory, or mapping their response onto the response categories provided [17].

### Sample

A total of 64 interviews were conducted in two rounds, from 2012 to 2013. The two rounds facilitated testing different versions of other questions in the study; the SRH question and its probes remained unchanged across the two rounds. An important goal of the Voices Heard Study, the larger research study in which this project was embedded, was to examine reporting differences for several survey questions across self-described categories of race and ethnicity [18]. Thus, members of the project team recruited sample members through connections they built with leaders in specific racial and ethnic communities, by visiting churches and community centers, by attending events sponsored by specific racial or ethnic groups (e.g., pow-wows), and by posting flyers at targeted locations in communities. We confined recruiting to the southern part of Wisconsin, mainly around Madison and Milwaukee. Our quota sampling strategy yielded nearly equal numbers of white, black, Latino, and American Indian participants crossed by gender (male, female), age (30–55 years, 56 years or more), and educational attainment (high school education or less, some college or more) (see Table 1).

**Table 1:** Number of Completed Interviews by Participants' Characteristics and Interviewing Round, *N* = 64

	Male				Female			
	30–55 years		56 years or more		30–55 years		56 years or more	
	HS	SC	HS	SC	HS	SC	HS	SC
Round 1								
Black	1	1	1	1	1	1	1	1
American Indian	0	2	1	1	1	1	1	1
White	1	1	1	1	1	1	1	1
Latino	1	1	1	1	1	1	1	1
Round 2								
Black	1	1	1	1	1	1	1	1
American Indian	1	1	1	1	1	1	1	1
White	1	1	1	1	1	1	1	1
Latino	1	1	1	1	1	1	1	1

For educational attainment, “HS” indicates high school degree or less and “SC” indicates some college or more

### Interviewing and transcription

Nine interviewers were recruited from the project staff, the University of Wisconsin Survey Center (UWSC), and the community. Interviewers received a full day of training on cognitive interviewing tailored for the study and were required to complete a practice interview before being certified. Interviewers and participants were matched on race/ethnicity and, for all cases except one, on gender. Interviewers conducted interviews at locations that were convenient for participants (e.g., public libraries, participants’ homes, and places on the UW campus). Interviews were primarily conducted in English, although eight participants elected to be interviewed in Spanish. On average, interviews took approximately an hour to complete (mean = 61.10 min, standard deviation = 20.17). Participants received a cash incentive of $30 for participating (this amount was increased to $50 to decrease the likelihood of canceled appointments). All of the interviews were audiotaped. In order to facilitate coding and analysis, interviews were transcribed verbatim and then entered on a question-by-question basis into Excel.

### Coding process

The four coders included three of the study authors and one staff member at UWSC, with backgrounds in sociology, survey methodology, cultural anthropology, and population health. (Two coders had also participated in the study as interviewers for some of the cases.) Our coding of the transcripts was inductive, iterative, and systematic. Consistent with the constant comparative method in grounded theory [12–14], we deployed a bottom-up process of identifying codes and categories as they emerged from the data and revising them with the addition of new data. Considering four to eight cases at a time, members of the research team independently developed codes for the text included in each turn-of-talk (one uninterrupted stream of talk from a participant) in Excel. We then met as a group to discuss our codes, building our coding scheme inductively through negotiated agreement among the coders. As we made changes to our evolving coding scheme, we returned to previously coded cases and recoded them to maintain consistency. After following this process with all 64 cases, we reconciled and revised both the coding scheme and coded cases to arrive at finalized versions of each.

### Analytic categories

The five analytic categories and their subdimensions derived from inductive coding of the cognitive interviews are numbered 1 through 5 below. We broke turns-of-talk by participants into segments of health factors (1) and descriptions and definitions of health (5). For each health factor, we coded its valence (2) and temporality (3). We also coded for whether participants made linkages among the health factors they mentioned using with a code for conditional health statements (4) and coded for the valence (2) of these conditional health statements.

1. **Types of health factors mentioned.** These include the health factors that participants mention thinking about when rating their health. These are defined in more detail in the Results and Table 2.
2. **Valence.** In psychology, valence refers to the degree of attraction or aversion an individual feels toward the specific objects or events in question [19]. In the current study, we define valence as the participant’s affective orientation to the health factor they mentioned based on what it implies about the quality of their current health. By coding for valence, we are able to highlight that participants who mention the same type of health factor may be using this information in different ways when formulating their rating of their health. For example, participants who mention their weight as a component of their physical state can do so with a positively-oriented valence, such as when they speak of deliberately losing weight, or with a negatively-oriented valence, such as when they speak of unintentionally gaining or needing to lose weight. We employed a rule in coding that to contextualize the valence of a given health factor, we included everything the participant said up to and including the current word or phrase in question.
  We coded several mutually exclusive dimensions of a health factor’s valence. Valence could be *positive* (e.g., “I have no illnesses”), *negative* (e.g., “I have several illnesses”), *neutral* (a point on the scale between positive and negative, e.g., “I’m about average”), or *ambivalent* (include both positive and negative valences, e.g., “I only have two things that’s not healthy about myself” in which the “not healthy” things are negative but the “only” is positive in terms of mitigating the negativity). We also coded for when the valence of a health factor was *not discernible*, that is, the factor is about the participant’s health but with a level of abstraction that does not reveal their orientation to the health factor, e.g., “How much I exercise” compared to “I don’t exercise.”
3. **Temporality of health factors.** Another way our study considers how participants formulate their health ratings is by coding the relative point in time to which the health factor refers: past, present, past to present, or conjecturing. Using health conditions as an example, a mention of *past* health conditions would be “I had a heart attack” and *present* would be “I have diabetes.” *Past to present* refers to a continued state over time, such as “I have been in pain for so many years.” Participants also often *conjecture* about something that does not exist at present, such as “if I didn’t have diabetes.”
4. **Conditional health statements.** By attending to how participants describe formulating their answers to the SRH question, we identified patterns in which some health factors are conditional on one another, meaning that the presence or absence of one health factor depends on another. We identified two types of conditional health statements. A *cascade* occurs when participants link two or more health factors together (often with a conjunction) in such a way that the presence of one factor causes the other, e.g., “if I lost weight [then] I would be able to say good or pretty good.” A *contrast* occurs when participants link two health factors together so that they juxtapose one another (e.g., “I do have some medical issues, but other than that, my health is really good”).
5. **Descriptions and definitions of health.** In addition to the multiple types of health factors participants mentioned, they also gave various types of descriptions and definitions of health when asked what they were thinking about when rating their health. First, participants often recapitulated their response to the SRH question by making statements that linked actual (e.g., “excellent” or “good”) or hypothetical (e.g., “fine”) response categories as adjectives with the subjects “I” or “my health” and forms of the verbs “to be” and “to feel.” We refer to these type of phrases as *adjective descriptions*: statements about one’s health that use an actual or hypothetical response category as an adjective to describe the participant’s health (e.g., “I guess I’m pretty good,” “I feel fine”). *Global statements* refer to phrases such as “overall,” “everything,” “in general” used by participants when describing what they were thinking about when rating their health. *Health definitions* are statements in which the participant provided some parameters for what they meant by health, but in an abstract way (e.g., “just my health overall,” “I was thinking about my physical health,” or “how I feel”). *Evaluative statements* are those in which the participants elaborated on a previous health factor they mention with an assessment of it (e.g., “that was scary,” “it’s really hard”). *Normative statements* are those in which participants implicitly or explicitly identified the commonality of their situation with others (e.g., “there’s always going to be stress” and “[pains] come along with age”).

### Analytic strategy

Except where otherwise noted, we report the presence of a given coding category at the level of the participant; for example, in describing the proportion of participants who mentioned at least one health condition. We performed a series of bivariate cross-tabulations, crossing each code by a sociodemographic characteristic (race/ethnicity, gender, age, or education); for example, health conditions (any vs. none) by race/ethnicity. We report two-tailed significance tests using Fisher’s exact test to account for small sample sizes and zero cells to highlight differences in each type of code across groups. Because of our small sample size, we also use the convention of noting when relationships are marginally significant at the *p* < .10 level. However, we note that our analysis of differences across groups is exploratory since the analysis leads to multiple statistical tests and the data are from a relatively small convenience quota sample.

## Results

Our qualitative analysis led to the identification and classification of various subdimensions of the following analytic categories, which we present in the Methods section: types of health factors mentioned, valence of health factors, temporality of health factors, conditional health statements, and general descriptions and definitions of health. We report the results for each of these analytic categories below.

### Types of health factors mentioned

Table 2 shows the percentage of participants who explicitly mentioned the listed types of health factors at least once while reporting what they were thinking about when rating their health. The most common health factor mentioned was health conditions: 70% of participants made reference to at least one health condition (e.g., “diabetes,” “no illness”). Examining subdivisions of health conditions, 41% of participants reported at least one specific health condition, 36% reported at least one nonspecific health condition (e.g., “my illnesses”), and 17% made reference to the absence of health conditions. Compared to younger participants, older participants were more likely to mention specific health conditions (*p* < .10) and less likely to mention an absence of health conditions (*p* < .05). Participants with at least some college were more likely to report the absence of health conditions compared to those with a high school education or less (*p* < .05).

**Table 2:** Percentage of Participants Mentioning Various Types of Health Factors at Least One Time

Type of Health Factor	Percentage	Examples from Transcript	Significance^a^			
			Race/Ethnicity	Gender	Age	Education
Health conditions (overall)	70%		–	–	–	–
Specific	41%	“I’m diabetic”	–	–	+	–
Nonspecific	36%	“my illnesses”	–	–	–	–
Absence	17%	“I have no medical conditions at all”	–	–	*	*
Health behavior	41%	“eating,” “I don’t exercise,” “trying to lose weight”	–	–	–	*
Health care practitioner or setting	25%	“I went to the doctor,” “I don’t go…”	–	–	–	–
Physical state	22%	“in good shape,” “overweight”	–	–	–	–
Comparative statements (overall)	19%		–	–	–	–
Compared present self to others	11%	“I was thinking about my husband…compared to him”	–	–	–	–
Compared present self to self at another time	11%	“I’m not in as good shape as I was 3 years ago”	–	–	–	–
Physical functioning	14%	“body working,” “ability to work,” “lazy”	*	–	–	–
Feel	14%	“how/what I feel” “I feel fine/good/great/sick”	–	–	–	–
Mental health	8%	“depression,” “I don’t have mental health issues”	–	–	+	–
Age	6%	“at the age of 51,” “I’m at an age…”	–	–	–	–
External factors	5%	“I’m a parent,” “family background,” “money for food”	–	–	–	–

^a^Fisher’s exact test for significant differences across groups (two-tailed). + *p* < .10, *p < .05

Forty-one percent of participants made at least one reference to a health behavior (e.g., “smoking,” “I don’t exercise”), and these mentions were more likely to occur for participants in the higher education group (*p* < .05). One quarter of participants made at least one reference to a health care practitioner or setting when rating their health. Twenty-two percent of participants mentioned their overall physical state, and 14% mentioned their physical functioning. Whites were more likely to discuss physical functioning compared to the other racial/ethnic groups (*p* < .05). Fourteen percent of participants used the word “feeling” to describe an internal state of their health (rather than a description of how they were thinking of something).

Comparative statements are defined in terms of explicit (e.g., “compared to others”) or implicit (e.g., “I’m not the healthiest”) comparisons that participants make between 1) their own health and the health of others (like the preceding examples) or 2) their health in the present and their health at other points in time (e.g., “I’m not in as good shape as I was 3 years ago,” “since I’ve hit my thirties I’ve started having health problems”). Overall, 19% of participants made some sort of comparative statement while describing how they rated their health. Among the specific types of comparative references, 11% of participants made comparisons to others, and 11% of participants compared their present self to themselves at other points in time (the past for all but one participant, who compared their present self to a hypothetical self, e.g., “I’m not as healthy as I could be”). It is interesting to note that no Latinos made any sort of comparative reference, although the difference across racial/ethnic groups did not reach conventional statistical significance levels.

In contrast to the several types of physical health factors participants mentioned, only 8% of participants explicitly mentioned mental health as figuring into their assessment of their health status. All five of these mentions of mental health occurred for older compared to younger participants (*p* < .10). Furthermore, only 6% of participants mentioned age as informing their rating. All four of these mentions of age were provided by male participants, although this difference across gender did not reach conventional statistical significance levels. Finally, only 3 participants (5%) mentioned external factors outside their immediate control as informing their health rating.

### Valence of health factors mentioned

We define valence as the participants’ affective orientation to the health factor they mentioned based on what it implies about the quality of their current health, and provide examples of each in the Analytic Categories subsection of the Methods section. Overall, 80% of participants mentioned at least one negatively valenced health factor, and a majority (59%) also mentioned at least one positively valenced health factor, indicating immediately that some participants are integrating disparate health information in forming their health assessment (Table 3). A majority (52%) of participants also mentioned at least one health factor with a “not discernible” valence, meaning that the health factor was mentioned in the abstract rather than indicating its impact on the participant’s health. Thirty percent of participants expressed at least one ambivalent or neutrally valenced health factor.

**Table 3:** Percentage of Participants with at Least One Health Factor Coded as Indicating a Negative, Ambivalent/Neutral, Positive, or Not Discernible Valence

		Significance^a^			
Valence	Percentage	Race/Ethnicity	Gender	Age	Education
Negative	80%	–	–	–	–
Ambivalent or Neutral	30%	*	+	–	–
Positive	59%	–	–	–	–
Not discernible	52%	–	–	–	–

^a^Fisher’s exact test for significant differences across groups (two-tailed). + *p* < .10, *p < .05

Men were more likely than women to mention at least one ambivalently or neutrally valenced health factor (*p* < .10). In addition, a difference by race/ethnicity was present in the data (*p* < .05): American Indians were less likely to mention ambivalently or neutrally valenced health factors and blacks and Latinos were more likely to mention these health factors, with whites in an intermediate position. 

Table 4 shows the percentage of mentions of each health factor that were coded with a negative, ambivalent/neutral, positive, or not discernible valence, showing the diversity in valence within particular kinds of health factors. The majority of mentions of (overall, specific, and nonspecific) health conditions, (overall and self) comparative statements, mental health, age, and external factors were negatively valenced, with pluralities of negatively valenced mentions for health behaviors, health care practitioners or settings, physical state, and feeling. The majority of mentions of absence of health conditions and comparisons to others were positively valenced, with large minorities or pluralities of positively valenced mentions of health behaviors, health care practitioners or settings, physical state, overall comparative statements, physical functioning, and feeling. Furthermore, some types of health factors were more likely to be formulated with a level of abstraction given the large proportions of “not discernible” mentions, including health behaviors, physical state, and physical functioning.

**Table 4:** Percentage of Health Factors Coded as Indicating a Negative, Ambivalent/Neutral, Positive, or Not Discernible Valence by Type of Health Factor

Type of Health Factor^a^	Valence (Percent)				Number of Times the Health Factor is Mentioned
	Negative	Ambivalent or Neutral	Positive	Not Discernible	
Health conditions (overall)	74%	6%	17%	3%	124
Specific	86%	6%	5%	3%	65
Nonspecific	90%	8%	0%	3%	40
Absence	0%	0%	95%	5%	19
Health behavior	40%	3%	32%	25%	63
Health care practitioner or setting	42%	8%	35%	15%	26
Physical state	42%	0%	29%	29%	24
Comparative statements (overall)	52%	13%	35%	0%	23
Self	83%	8%	8%	0%	12
Others	18%	18%	64%	0%	11
Physical functioning	11%	0%	48%	41%	27
Feel	31%	23%	31%	15%	13
Mental health	56%	11%	22%	11%	9
Age	50%	0%	25%	25%	4
External	60%	0%	20%	20%	5

^a^Rows sum to 100%

We further examined how the valence of health factors mentioned varied by the SRH response category selected (Table 5). Of the 64 participants, a plurality (44%) selected the middle category “good,” followed by the categories that surround it—“very good” (31%) and “fair” (19%). Very few participants selected “excellent” (6%) and no participants selected “poor.” Somewhat unsurprisingly, the percentage with at least one negatively valenced health factor decreased with better SRH (*p* < .05): The percentage of participants with at least one negatively valenced health factor was 100% for those answering “fair,” 86% for those answering “good,” 65% for those answering “very good,” and 50% for those answering “excellent.” Similarly, all participants reporting “excellent” health had at least one positively valenced health factor, and the percentage with at least one positively valenced health factor decreased with worse SRH (*p* < .01).

**Table 5:** Percentage of Participants with at Least One Health Factor Coded as Indicating a Negative, Ambivalent/Neutral, Positive, or Not Discernible Valence within Self-Rated Health Category Chosen

	Self-Rated Health Answer				
	Fair	Good	Very good	Excellent	
Overall					
Percentage	19%	44%	31%	6%	
Number of participants	12	28	20	4	
Valence^a^					
Negative (vs. none)	100%	86%	65%	50%	*
Ambivalent or neutral (vs. none)	17%	46%	20%	0%	+
Positive (vs. none)	25%	54%	80%	100%	**
Not discernible (vs. none)	25%	57%	65%	25%	+

^a^Fisher’s exact test for significant differences across groups (two-tailed). + *p* < .10, *p < .05, **p < .01

The results in Table 5 also highlight the integration of disparate aspects of health in answering SRH. For example, 25% of participants who said their health was "fair" still mentioned at least one health factor that was positively valenced with respect to the quality of their current health. The integration of disparate aspects is also evident in the percentage of participants who had at least one ambivalently or neutrally valenced health factor, which varied across the SRH response categories (*p* < .10). That these ambivalent/neutral mentions occurred for almost half (46%) of participants who answered “good” provides evidence that participants selecting “good” may be attempting to integrate disparate health information when selecting their response to the survey question. Finally, the results in Table 5 show that mentions of health factors with a “not discernible” valence also varied across SRH category (*p* < .10), with the largest percentages for respondents who reported their health to be “very good” or “good.”

### Temporality of health factors mentioned

The temporality analytic category captures information about the relative point in time to which a health factor refers. Overall, 97% of participants had at least one health factor that referred to the present, 23% had at least one factor referring to the past, 13% had a factor that spanned from past to present, and 19% had at least one conjecturing health factor (not shown). The temporality of health factors did not vary significantly by race/ethnicity, gender, age, or education, or by the response category chosen when answering the SRH question (not shown).

### Conditional health statements

Conditional health statements refer to when the presence or absence of one health factor depends on another; these can either cascade (e.g., “if X then Y”) or contrast (e.g., “X but Y”). Sixty-four percent of participants had at least one conditional health statement; 34% had at least one cascade, and 36% had at least one contrast (Table 6). The only marginally significant difference across groups, however, was that those with some college education or more were more likely to have a cascade than those with a high school education or less (*p* < .10).

**Table 6:** Percentage of Participants with at Least One Conditional Health Statement

	Percentage	Significance^a^			
		Race/Ethnicity	Gender	Age	Education
Conditional health statement	64%	–	–	–	–
Cascade	34%	–	–	–	+
Contrast	36%	–	–	–	–

^a^Fisher’s exact test for significant differences across groups (two-tailed). + *p* < .10, *p < .05

We also coded the valence of the entire conditional health statement. There were 52 unique conditional health statements (some participants had more than one); half were cascades and half were contrasts (not shown). Nineteen of the 26 unique cascades were negative. Coding the valence of the conditional health statements also revealed another way in which the integration of different aspects of health is displayed by participants—21 of the 26 unique contrasts were ambivalent, integrating both positive and negative health factors in formulating an assessment of one’s health (not shown).

### Descriptions and definitions

In addition to the various types of health factors, participants expressed various descriptions and definitions of health when asked to describe what they were thinking about when rating their health (Table 7). Forty two percent of participants had at least one adjective descriptions (e.g., “I guess I’m pretty good,” “I feel fine”) when asked what they were thinking about when they rated their health. Fourteen percent had at least one global statement as part of their response (e.g., “overall,” “everything,” “in general”). Thirteen percent of participants had at least one health definition, providing some abstract parameters for what they were thinking about when rating their health (e.g., “just my health overall,” “I was thinking about my physical health,” or “how I feel”). Eight percent of participants had at least one evaluative statement that assessed a previous health factor mentioned (e.g., “that was scary,” “it’s really hard”). Finally, 6 % of participants had at least one normative statement in which they implicitly or explicitly identify the commonality of their situation with others (e.g., “there’s always going to be stress,” “[pains] come along with age”). None of descriptions and definitions vary across the sociodemographic groups.

**Table 7:** Percentage of Participants with at Least One Description or Definition

Valence	Percentage	Significance			
		Race/Ethnicity	Gender	Age	Education
Adjective description	42%	–	–	–	–
Global statement	14%	–	–	–	–
Health definition	13%	–	–	–	–
Evaluative statement	8%	–	–	–	–
Normative statement	6%	–	–	–	–

## Discussion

In order to provide a more comprehensive understanding of how SRH functions as a measure of health, the two main goals for this study were to provide a qualitative description of how participants rate their health when asked to do so and to examine whether features of this response process vary across sociodemographic groups. We used cognitive interviewing to elicit descriptions of what participants consider when rating their health and qualitative analysis to identify both *which* health factors they take into account as well as *how* they take these health factors and components of the SRH question into account when rating their health. Participants do not simply list the health factors they consider, but often cast their answers in a way that reveals how those health factors and components of the question are experienced, conceptualized, interpreted, and integrated to formulate answers to SRH.

Our qualitative analysis led to the identification and classification of various subdimensions of the following analytic categories: types of health factors mentioned, valence of health factors, temporality of health factors, conditional health statements, and general descriptions and definitions of health. We consider the findings from each of these analytic categories below.

We argue for the merit in adding to the body of studies that examine the health factors respondents consider when rating their health, as doing so across time, place, and groups bolsters previous findings and documents health factors previously undescribed. In addition to replicating some of the types of health factors mentioned in prior qualitative studies, such as mentions of health conditions and physical functioning [8], one new health factor emerged from our study that was not identified previously. Comparisons to relevant others have been documented as one of the factors participants consider when formulating their response to SRH [7, 8], and the current study highlights another relevant comparison that some participants make—a comparison to themselves in the past, which has been proposed as contributing to health ratings [3] but was not reported in prior qualitative studies as something respondents state as a consideration when rating their health.^1^ An additional interesting finding from our analysis is the health factors participants did *not* mention taking into account when formulating their SRH answer. In particular, stress, spirituality, mental health, age, and external factors like family background or socioeconomic circumstances were rarely or not mentioned by participants.

Our contribution of the characterizations of *how* participants formulate SRH is an important extension of prior research. To the best of our knowledge, this is the first study to systematically describe and analyze the analytic categories of valence, temporality, conditional health statements, and descriptions and definitions of health as important components of participants’ ratings of health. Importantly, our coding of valence relies on our perception of the participant’s orientation to the health factor mentioned as displayed in the cognitive interview, rather than researchers’ a priori evaluation of what constitutes a positive or negative component of health [8]. We note that future research can use valence in order to make distinctions among participants who mention the same type of health factor yet use information in different ways when formulating their rating of their health; we could not examine group differences in this process due to sample size. In addition, we argue that in order to understand what a health factor means for a participant’s health, probing needs to focus on this when the valence of the health factor is not discernible (“how much I exercise” vs. “I exercise a lot/not at all”). This extends beyond SRH to other studies that seek to map out the dimensions of complex concepts with cognitive interviewing.

In our characterization of how participants formulate SRH, we also documented conditional health statements, which took the form of cascading (sequentially linked) health factors or contrasts that largely integrated disparately valenced health factors, both of which were previously undescribed in the literature. Overall, we observed various ways in which seemingly disparate health factors are integrated to formulate an answer: negatively valenced health factors reported with positive self-rated health (and vice versa), ambiguous or neutrally valenced health factors, and two types of conditional health statements: cascades and contrasts.

In addition, our study highlighted the temporal dimensions that underlie participants’ health ratings, mainly considering the present, but also the past, trajectories from past to present [3], and conjecturing about a hypothetical future self. The descriptions and definitions of health that occur when participants are asked to describe what they are thinking about when they rate their health were previously undescribed in the qualitative studies in which respondents report what they think about when rating their health. In our inductive coding we observed four categories of descriptions or definitions that participants use: adjective descriptions, global statements, health definitions, evaluative statements, and normative statements.

We argue that it is important to understand all the components—the *which* and the *how*—underlying SRH in order to understand its validity as a measure of health for research and monitoring purposes. As we see in this study, two participants who report the same SRH and the same health condition may have different valences or temporality for that health factor, and different conditional relationships with other health factors; treating the SRH answers reported in the survey (and the reported health condition) as the same for these two hypothetical respondents ignores this heterogeneity. In addition, participants across the range of answer categories appear to integrate disparate aspects of their health through the use of contrasting conditional health statements, ambiguously valenced health factors, and health factors with valences that do not align with their health rating (e.g., reporting “excellent” health and a negatively valenced health factor). Thus, underlying ratings of health is a web of interrelated and sometimes conflicting components of health. In terms of implications for researchers and analysts that use SRH, this tells us that 1) SRH is doing what researchers assume it is doing in terms of prompting participants to summarize their health [8]—although what is considered and by whom varies across sociodemographic groups—and 2) prior research is incomplete with respect to representing what underlies ratings of health, as how these health factors are perceived and integrated to formulate an answer is not captured in prior research. Thus, the results of prior and future studies that use SRH as a measure of health should be interpreted with this complexity and heterogeneity in mind. More specifically, it indicates that treating SRH as proxy for more objective health without adjusting in some way for this complex and heterogeneous response process can lead to errors in measurement and interpretation, as the measure then conflates both the more objective health factors that inform the rating and participants’ evaluative response processes [4].

Although the characterizations of “how” participants formulate their SRH answers are an important contribution, we sought to supplement these with examination of differences across race, ethnicity, gender, education, and age. Because of the small convenience quota sample, however, the results are clearly exploratory and meant to highlight new avenues for future research, particularly since 1) we do not have the statistical power to confidently detect group differences and 2) we do not have controls for “more objective” health that would help to delineate whether the differences across groups in the SRH response process we observe here are due to group differences in more objective health or differences in evaluative frameworks. Furthermore, a focus on between-group differences should be supplemented with a focus on intersecting systems of identity and oppression which cannot be examined in this small convenience quota sample with two respondents in each “cell” that intersects race/ethnicity, gender, age, and education [20, 21].

Limitations noted, however, some of the findings of group differences in this study are consistent with patterns from prior qualitative and quantitative studies. For example, differences by age and education in the types of health factors mentioned correspond to patterns in health disparities by age and education, with younger and more educated persons having better health outcomes (in this case, reporting an absence of health conditions or not reporting the presence of health conditions) [22]; this finding for age is consistent with prior qualitative research [8, 11]. White participants being more likely than other groups to mention physical functioning as part of how they rate their health is also consistent with prior qualitative research by Krause and Jay [8].

In addition, some of the findings of group differences were counter to expectation or highlight avenues for future research. Mental health was more likely to be mentioned for older compared to younger participants, underscoring an interesting juxtaposition with prior research that finds no differences in age in considering mental factors to rate one’s health [7, 11]. Similarly, participants with more education were more likely to mention health behaviors when rating their health, counter to the findings of Krause and Jay [8], who found that respondents with lower levels of education were more likely to mention health behaviors. This inconsistency could be sorted out in a larger study in which the valence of health behaviors is considered. Further, those with some college education or more were more likely to have a cascade than those with less education. In a synthesis of research on cross-cultural cognitive interviewing, Willis [23] discusses a few studies in which respondents with lower educational attainment have more indicators of difficulty in answering cognitive interviewing probes. Although speculative, we find it plausible that those with less education are more likely to simply list health information when asked what they were thinking of when rating their health given the metacognitive burden of performing this task, while those with more education are more likely to make linkages among health factors in the form of a cascade.

Regarding race/ethnicity, no Latinos made any sort of comparative reference, aligning with previous research which shows that Latinos have a more collectivist orientation (prioritizing group over individual goals) compared to, for example, non-Hispanic whites [24]. We posit that a collectivist orientation may prevent comparisons to others when rating one’s health—or explicitly mentioning such comparisons—if such comparisons are perceived to be a source of discord by invoking hierarchy or individuation. In addition, future work should examine why American Indians, a population previously undescribed with respect to what underlies their SRH, may be less likely to reference ambivalently or neutrally valenced health factors, as well as why blacks and Latinos may be more likely to do so.

All four mentions of age as a health factor were provided by male participants. Although the number is too small to draw conclusions about group differences, it is plausible that women may be less inclined to explicitly mention age in this and other contexts given the gendered nature of ageism in the US [25]. It is also interesting to note that men were more likely to mention ambivalently or neutrally valenced health factors. This finding highlights one pathway through which the apparent “health optimism” of men in the US relative to women (at least prior to older ages) might occur [26]. We posit that with poorer objective health, men may rate their health better than do women because men interpret the health factors through a lens of ambivalence or neutrality as opposed to purely negative.

This study builds upon prior work by characterizing how participants formulate their health ratings more holistically, identifying several components of rating health that researchers should attend to beyond the types of health factors participants consider. Our analytic approach itself is an important contribution to the analysis of cognitive interviews and transcribed talk more generally. We coded participants’ cognitive interviews in an inductive, iterative, and systematic process, and include in our analysis all parts of their answers to the probes following SRH. Our group consensus approach was particularly useful in examining words and phrases with multiple meanings, and allowed us to vet assumptions and inferences about what participants might have meant by something they said. We recommend that the process we used to code utterances about SRH for this study be used in other studies of SRH with larger samples.

Some limitations to note include that the interviewing logistics—in which participants traveled to be interviewed—precluded recruiting the very ill. We had very few participants reporting “excellent” health and no participants reporting “poor” health, so we are missing a comprehensive description of what underlies SRH at the extremes of the rating scale. In addition, differences observed across race/ethnicity, gender, age, and educational attainment might be due to confounding factors such as occupation, household income, and access to health care. Furthermore, the results from a Wisconsin convenience sample may not be generalizable to other regions and sociodemographic groups. Finally, the cognitive interviewing process itself may influence the descriptions participants provide, as the answers obtained are dependent on what the probes ask participants to do and which parts of the probes participants attend to. Some participants may be more adept than others in verbally delineating their SRH response process in a follow-up—retrospective—probe, and it is likely that participants attend to different facets of the follow-up probe in the same way they attend to different facets of SRH. Thus, the analysis presented here is a more direct observation of the SRH response process than previously described, but it is not complete.

## Conclusion

Overall, this study serves to provide a more direct and comprehensive description of the SRH response process, explicating how participants formulate their answers to SRH by attending to *which* health factors participants take into account and *how* participants take these health factors and components of the SRH question into account when evaluating and rating their health, with particular attention to variations across several sociodemographic characteristics. Studies such as this may be useful in deciding whether and when SRH, a subjective assessment, can be used to examine group differences in (objective) health, by delineating the components that underlie health ratings that vary across groups. The methods employed here (cognitive interviewing and grounded theory coding in an inductive, iterative, and systematic procedure) can be combined and employed in other domains to examine what underlies ratings of other subjective assessments that are used in survey research.

## Abbreviation

SRH: Self-rated health

## Footnotes

1 Groves and colleagues [6] examined whether participants made comparisons to others or themselves in the past using closed-ended questions that prompts participants to explicitly make this comparison. The current study documents comparisons to oneself in the past deriving from information revealed spontaneously in participants’ descriptions of their response process.
